# Difference in distribution profiles between CD163+ tumor-associated macrophages and S100+ dendritic cells in thymic epithelial tumors

**DOI:** 10.1186/s13000-014-0215-7

**Published:** 2014-12-14

**Authors:** Mutsuko Omatsu, Toshiaki Kunimura, Tetsuya Mikogami, Akira Shiokawa, Tomoko Nagai, Atsuko Masunaga, Akihiko Kitami, Takashi Suzuki, Mitsutaka Kadokura

**Affiliations:** Department of Clinico-diagnostic Pathology, Showa University Northern Yokohama Hospital, 35-1 Chigasaki-chuo, Tsuzuki-ku, Yokohama, 224-8503 Japan; Department of Clinico-diagnostic Pathology, Showa University School of Medicine, Tokyo, Japan; Respiratory Disease Center, Showa University Northern Yokohama Hospital, Yokohama, Japan; Division of Thoracic and Cardiovascular Surgery, Department of Surgery, Showa University School of Medicine, Tokyo, Japan

**Keywords:** Thymic carcinoma, Thymoma, Tumor-associated macrophages, CD163, Dendritic cells, S100

## Abstract

**Background:**

In a number of human malignancies, tumor-associated macrophages (TAMs) are closely involved in tumor progression. On the other hand, dendritic cells (DCs) that infiltrate tumor tissues are involved in tumor suppression. However, there have been very few reports on the distribution profiles of TAMs and DCs in thymic epithelial tumors. We examined the difference in the distribution profiles between TAMs and DCs in thymoma and thymic carcinoma.

**Methods:**

We examined 69 samples of surgically resected thymic epithelial tumors, namely, 16 thymic carcinomas and 53 thymomas, in which we immunohistochemically evaluated the presence of TAMs using CD68 and CD163 as markers and DCs using S100 as the marker in tumor tissue samples in comparison with normal thymic tissues.

**Results:**

The percentage of samples with a large number of CD68+ TAMs was not significantly different between thymic carcinoma and thymoma (7/16 versus 16/53, *p* = 0.904). However, the percentage of sample with a large number of CD163+ TAMs was significantly higher in thymic carcinoma than in thymoma (15/16 versus 34/53, *p* = 0.024). In contrast, the percentage of samples with a large number of S100+ DCs was significantly lower in thymic carcinoma than in thymoma (2/16 versus 23/53, *p* = 0.021).

**Conclusions:**

To the best of our knowledge, we are the first to show a high percentage of CD163+ TAMs and a low percentage of S100+ DCs in thymic carcinoma samples, and our findings may provide an idea for future targeted therapeutic strategies for thymic carcinoma using antibodies that inhibit monocyte differentiation to TAMs, thereby skewing TAMs differentiation toward DCs.

**Virtual Slides:**

The virtual slide(s) for this article can be found here: http://www.diagnosticpathology.diagnomx.eu/vs/13000_2014_215

## Background

Macrophages that infiltrate tumor tissues are referred to as tumor-associated macrophages (TAMs) and are closely involved in tumor progression by inducing angiogenesis, immunosuppression, and invasion [1]. The protumoral role of TAMs is supported by many clinical studies of carcinomas, including breast, prostate, endometrial, and bladder carcinomas, and malignant lymphomas, which showed a correlation between a large number of macrophages and poor prognosis [2,3]. On the other hand, dendritic cells (DCs) that infiltrate tumor tissues are involved in tumor suppression via immune responses. A large number of DCs is related to better survival in a variety of malignant tumors such as melanoma, breast carcinoma, hepatocellular carcinoma, and lung adenocarcinoma [4].

Thymic epithelial tumors are rare mediastinal tumors and can be classified into thymoma and thymic carcinoma. Compared with other organs, the human thymus is a lymphoepithelial organ, and macrophages and DCs, as well as epithelial cells, are the cellular components; however, there have been very few reports [5] concerning TAMs and DCs in thymic epithelial tumors. Here, we immunohistochemically examined tumor tissue samples to characterize TAMs and DCs in thymoma and thymic carcinoma by comparing them with those in normal thymic tissues.

## Methods

### Patients and samples

**Figure 1:**
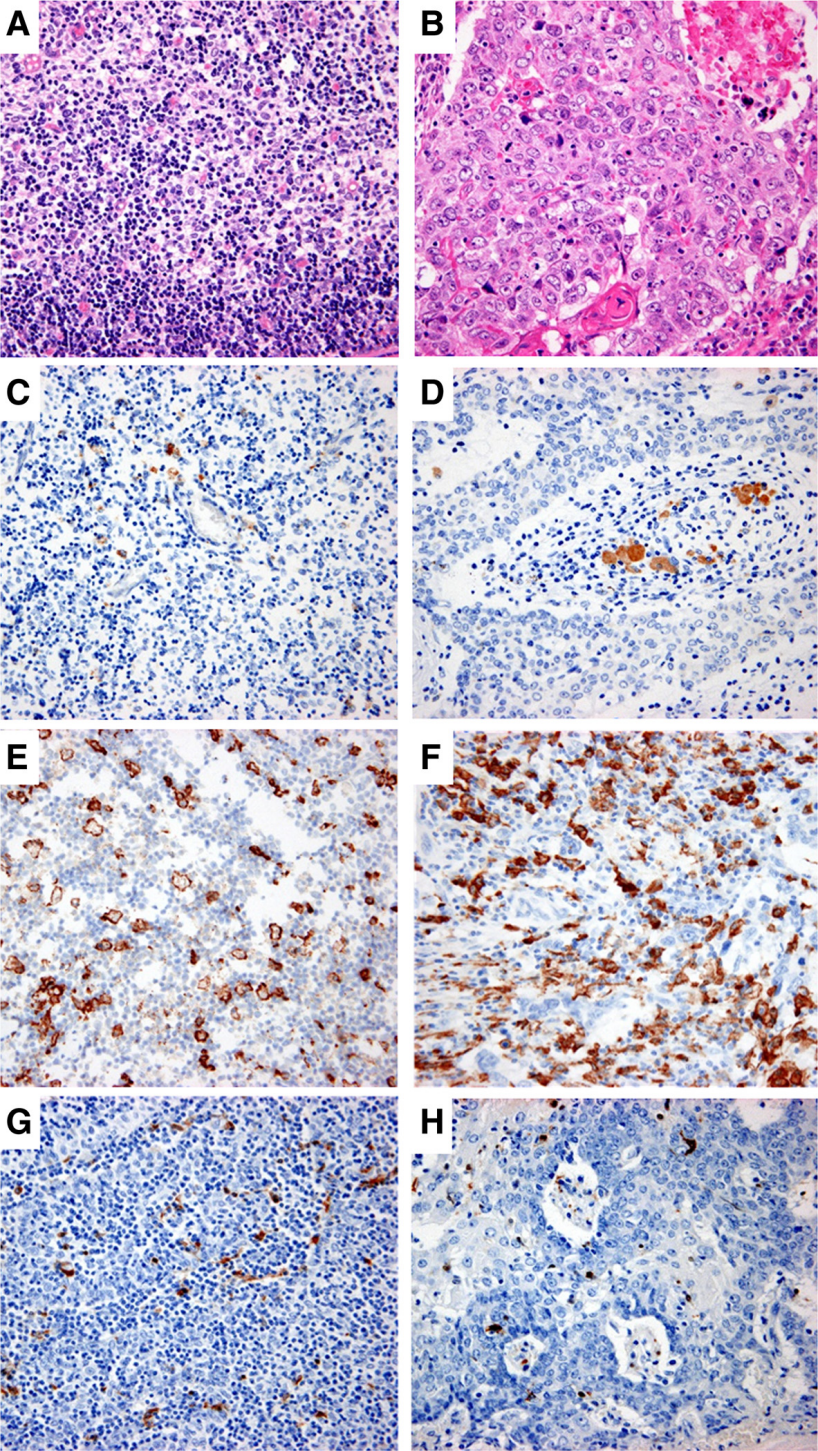
**Photomicrographs of thymic lesions. A**: High-power view of type B2 thymoma showing polygonal, medium-sized tumor cells associated with lymphocytes (H&E staining). **B**: High-power view of thymic carcinoma showing large polyhedral tumor cells with eosinophillic cytoplasm and apparent nuclei (H&E staining). **C**: High-power view of type B2 thymoma showing 1.50% of cells immunohistochemically stained positive for CD68 (immunohistochemistry). **D**: High-power view of thymic carcinoma showing 1.16% of cells immunohistochemically stained positive for CD68 (immunohistochemistry). **E**: High-power view of type B2 thymoma showing 6.95% of cells immunohistochemically stained positive for CD163 (immunohistochemistry). **F**: High-power view of thymic carcinoma showing 35% of cells immunohistochemically stained positive for CD163 (immunohistochemistry). **G**: High-power view of type B2 thymoma showing 2.83% of cells immunohistochemically stained positive for S100 (immunohistochemistry). **H**: High-power view of thymic carcinoma showing 1.30% of cells immunohistochemically stained positive for S100 (immunohistochemistry).

In this study, we examined samples from 69 patients diagnosed and treated for primary thymic epithelial tumors at Showa University Northern Yokohama Hospital, Showa University Hospital, and Showa University Fujigaoka Hospital from August 2003 to June 2014. The samples were obtained by surgical resection without neoadjuvant therapy from 16 patients with thymic carcinoma (Figure 1B) (10 males and 6 females; age range, 34–79 years, including no myasthenia gravis patient) and 53 patients with thymoma (Figure 1A) (28 males and 25 females; age range, 30–83 years, including 9 myasthenia gravis patients). Follow-up data were available for 62 patients, including 9 with thymic carcinoma and 53 with thymoma. At the time of analysis, only one of the 9 patients with thymic carcinoma died of disease, one alive with recurrence, and 7 with alive without recurrence, the other hand, only one of the 53 patients with thymoma died of disease, three alive with recurrence and 49 alive without recurrence (Table 1). All of the tissue samples were fixed in 20% formalin, routinely processed, embedded in paraffin wax, cut into 3-μm-thick sections, and stained with hematoxylin and eosin (HE). Thymoma was the diagnosis when immature T cell markers, such as CD99 and TdT, were stained in the underlying lymphocytes, and thymic carcinoma was the diagnosis when CD5 and c-kit were stained in epithelial cells. This study was approved by the Ethics Committee of Showa University Northern Yokohama Hospital (No.#1406-02).

**Table 1:** **Characteristics of patients with thymoma and thymic carcinoma**

**Characteristic**		**Thymoma**	**Thymic carcinoma**
Patient, n		53	16
Gender, male/female		28/25	10/6
Mean age, yr ± SD		52 ± 13.6	67 ± 12.2
Stage			
	I	30	0
	II	17	7
	III	5	4
	IV	1	3
	Unknown	0	2
Pathology		A: 3	SCC: 16
		AB: 14	
		B1: 17	
		B2: 14	
		B3: 5	

Stage: Based on WHO classification.

SCC: squamous cell carcinoma.

### Immunohistochemical staining

A list of antibodies with their clones, sources, dilutions, antigen retrieval methods, and incubation durations is presented in Table 2. After endogenous peroxidase activity was inhibited in the prepared sections using hydrogen peroxide solution, the sections were incubated with the appropriate primary antibody. A secondary antibody was raised against biotinylated immunoglobulin and conjugated with avidin-horseradish peroxidase (HRP). The staining of each sample was visualized using a Ventana I-View DAB universal kit (Roche, Tokyo, Japan). The antibody-antigen reaction was enhanced using copper sulfate, after nuclear staining with Mayer’s hematoxylin. Positive controls for the antibodies were prepared in accordance with the manufacturer’s instruction.

**Table 2:** **Antibodies used for immunohistochemical analysis**

**Antibody**	**Clone**	**Source**	**Dilution**	**Antigen retrieval**	**Incubation (min)**
CD68	KP-1	DAKO	Prediluted	Protease (0.5 unit/ml) for 8 min	32
CD163	10D6	Leica NOVO	1:100	Heat treatment of EDTA for 30 min	32
S100 protein	4C4.9	VENTANA Roche	Prediluted	Not required	16

### Evaluation of immunohistochemical staining

For the enumeration of CD68+, CD163+ TAMs and S100+ DCs, ten representative fields were examined at high-power magnification (400×). Only CD68+, CD163+ cells showing a macrophagic morphology, and only S100+ cells showing a dendritic morphology were counted. The percentages of positively stained TAMs and DCs were calculated in relation to overall cellularity [6-8] using hard-copy photomicrographs. The cells were counted in duplicate by two different pathologists (M.O. and T.K.) without knowledge of the patients’ clinical data. The obtained data were then averaged and defined as the TAM and DC contents of the samples. Normal thymic samples obtained distal to the tumor site from 34 patients (18 males and 16 females; age range, 38-83 years) were used as the controls. The median percentage of markers-positive macrophages or DCs in normal thymic samples was used as the cut-off point to categorize the thymic epithelial tumor samples into the group with a low percentage of marker-positive TAMs of DCs and that with a high percentage of these cells.

### Statistical analysis

The variables measured in this study were tested for associations using the Chi-squared test Fisher’s exact probability test and Mann-Whitney’s U test. A *p* value <0.05 was considered statistically significant.

## Results

### CD68+ TAM

The percentage of CD68+ macrophages varied from 0.11% to 5.29% with a median of 1.31% in normal thymic samples. The percentage of CD68+ TAMs varied from 0.13% to 7.54% with a median of 0.96% in thymoma samples (Figure 1C), and 0.00% to 7.91% with a median of 1.08% in thymic carcinoma samples (Figure 1D). Regarding the positivity for CD68, the thymoma and thymic carcinoma samples were categorized into two groups on the basis of the 1.31% cut-off point that indicates the median in normal thymic samples. A high percentage of CD68+ TAMs was observed in 43.8% (7/16) of thymic carcinoma samples and 30.2% (16/53) of thymoma samples (including 3 type A, 3 type AB, 5 type B1, 2 type B2 and 3 type B3), which were not statistically significantly different (*p* = 0.904) (Table 3a).

**Table 3:** **Percentage of samples showing CD68, CD163, and S100 expression in thymoma and thymic carcinoma**

		**Low**	**High**	***p-value***
(a) CD 68	Thymoma	37	16	0.904
	(A/AB/B1/B2/B3)	(0/11/12/12/2)	(3/3/5/2/3)	
	thymic carcinoma	9	7	
(b) CD 163	Thymoma	19	34	0.024
	(A/AB/B1/B2/B3)	(1/2/11/4/1)	(2/12/6/10/4)	
	thymic carcinoma	1	15	
(c) S100	Thymoma	30	23	0.021
	(A/AB/B1/B2/B3)	(2/10/12/5/1)	(1/4/5/9/4)	
	thymic carcinoma	14	2	

The median percentages of marker-positive TAMs or DCs in normal thymic samples, (1.31% for CD68, 7.28% for CD163, and 1.50% for S100), were used as the cut-off points to categorize the samples into the group with a low percentage of marker-positive TAMs or DCs ant that with high percentage of these cells.

The correlations between percentage of CD68+ TAMs and the stage categories in thymoma and thymic carcinoma were shown in Table 4, which were not statistically significantly different (*p* = 0.853 and *p* = 0.262) (Table 4a).

**Table 4:** **The correlations between percentage of samples showing CD68, CD163, S100 expression and the stage categories in thymoma and thymic carcinoma**

		**Thymoma**			**Thymic carcinoma**		
**(a) CD68**	**Stage**	**Low**	**High**	***p-value***	**Low**	**High**	***p-value***
	I	21	9	0.853	0	0	0.262
	II	11	6		5	2	
	III	4	1		2	2	
	IV	1	0		1	2	
		**Thymoma**			**Thymic carcinoma**		
**(b) CD163**	**Stage**	**Low**	**High**	***p-value***	**Low**	**High**	***p-value***
	I	10	20	0.754	0	0	0.138
	II	7	10		0	7	
	III	2	3		2	4	
	IV	0	1		1	2	
		**Thymoma**			**Thymic carcinoma**		
**(c) S100**	**Stage**	**Low**	**High**	***p-value***	**Low**	**High**	***p-value***
	I	19	11	0.279	0	0	0.691
	II	8	9		6	1	
	III	3	2		4	0	
	IV	0	1		2	0	

Stage: Based on WHO classification.

2 cases of thymic carcinoma were in unkown stage.

### CD163+ TAM

The percentage of CD163+ macrophages varied from 4.95% to 10.4% with a median of 7.28% in normal thymic samples. The percentage of CD163+ TAMs varied from 4.38% to 23.98% with a median of 9.23% in thymoma samples (Figure 1E), and 6.53% to 33.26% with a median of 14.55% in thymic carcinoma samples (Figure 1F). Regarding the positivity for CD163, the thymoma and thymic carcinoma samples were categorized into two groups on the basis of the 7.28% cut-off point that indicates the median in normal thymic samples. A high percentage of CD163+ TAMs was observed in 93.8% (15/16) of thymic carcinoma samples and 64.2% (34/53) of thymoma samples (including 2 type A, 12 type AB, 6 type B1, 10 type B2 and 4 type B3), which were statisfically significantly different (*p* = 0.024) (Table 3b). Moreover, the percentage of samples with a large number of CD163+ TAMs was higher in the thymic carcinoma samples than in the thymoma samples.

The correlations between percentage of CD163+ TAMs and the stage categories in thymoma and thymic carcinoma were shown in Table 4, which were not statistically significantly different (*p* = 0.754 and *p* = 0.138) (Table 4b).

### S100+ DCs

The percentage of S100+ DCs varied from 0.41% to 5.02% with a median of 1.50% in normal thymic samples, 0.13% to 4.45% with a median of 1.28% in thymoma samples (Figure 1G), and 0.06% to 3.99% with a median of 0.79% in thymic carcinoma samples (Figure 1H). Regarding the positivity for S100, the thymoma and thymic carcinoma samples were categorized into two groups on the basis of the 1.50% cut-off point that indicates the median in normal thymic samples. A high percentage of S100+ DCs was observed in 12.5% (2/16) of thymic carcinoma samples and 43.4% (23/53) of thymoma samples (including 1 type A, 4 type AB, 5 type B1, 9 type B2 and 4 type B3), which were statistically significantly different (*p* = 0.021) (Table 3c). Moreovers, the percentage of samples with a large number of S100+ DCs was higher in the thymoma samples than in the thymic carcinoma samples.

The correlations between percentage of S100+ DCs and the stage categories in thymoma and thymic carcinoma were shown in Table 4, which were not statistically significantly different (*p* = 0.279 and *p* = 0.691) (Table 4c).

## Discussion

Macrophages are found in the cellular microenvironment of many carcinomas, and these TAMs represent a heterogeneous population of functionally distinct cells [2] that may affect the neoplastic process. Different phenotypes, as well as different cytokine secretion profiles, have suggested a distinction between ‘proinflammatory M1’ and ‘immunosuppressive M2’ macrophages [9]. Although it is now acknowledged that the binary M1/M2 model is oversimplified [10,11] and that there is a spectrum of intermediate macrophage phenotypes in response to various local microenvironmental signals [12,13], TAMs most often seem to exhibit M2 features [14]. However, at present, there is no single marker for macrophage polarization [15].

In the tumor microenvironment, TAMs play a key role in carcinoma-associated inflammation and affect the progression and prognosis of various tumor types [16,17] other than colorectal-gastric carcinoma and osteosarcoma [1], and a dense macrophage infiltrate is associated with enhanced nodal metastases, distant metastases, and reduced recurrence-free survival [18]. On the other hand, there have been only a few reports comparing the TAMs in malignant tumors with those in benign tumors arising in the same organs. In ovarian tumors, the number of CD68+, CD163+ TAMs is reported to show a stepwise increase from benign, borderline to malignant [19]. In thyroid tumors, the number of CD68+ TAMs is also higher in papillary carcinoma than in follicular adenomas [20]. In thymic tumor, although there has been no report comparing TAMs between thymoma and thymic carcinoma, in this study, we confirmed previous findings for thymic epithelial tumor. Although the percentage of CD68+ TAMs was not significantly different between thymoma and thymic carcinoma, in thymic carcinoma, which is associated with more frequent invasive growth and distant metastases, a higher percentage of CD163+ TAMs was found. These observations suggest that malignant tumors harbor a higher percentage of CD163+ TAMs than benign tumors in their microenvironment, which is a reasonable finding considering the roles of TAMs in malignant tumors.

CD68 is a glycoprotein used as a macrophage marker but is nonspecific. On the other hand, CD163 is a member of the scavenger receptor family and is specific for macrophages [7]. Immunohistochemical studies of TAMs demonstrated the superiority of CD163 over CD68 in predicting the clinical outcome [17-19]. We found a higher percentage of CD163+ TAMs than of CD68+ TAMs in both thymoma and thymic carcinoma samples, which was consistent with previous observations in Hodgkin’s lymphoma [7], malignant melanomas [20], and leiomyosarcomas [21]. Thus, the use of CD68 may lead to underestimation of the true percentage of TAMs [18].

DCs, i.e., specialized antigen-presenting cells, play a critical role in innate and adaptive immune responses [22]. In the tumor microenvironment, DCs are the most potent antigen-presenting cells that induce antigen-specific immune responses by engulfing dying tumor cells [23]. Among several DC markers, many investigators have used S100 as a valuable marker of DCs, because S100 show adequate immunohistochemical staining on paraffin tissue sections [24]. Several studies showed that the number of S100+ DCs in colon carcinoma [25], human gastric carcinoma [26], and esophageal carcinoma [27] negatively correlates with lymph node metastases, size of tumor, and survival time, that is, the larger the number of DCs, the better the patient’s prognosis. In addition to these clinicopathological parameters, a recent study of uterine endometrioid adenocarcinoma demonstrated the inverse correlation between a large number of S100+ DCs and the histological grade for malignant potential [28].

S100 can also be used as a useful marker of thymic DCs [29-31]. In thymic tumor, although there has been no report showing the relationships between the percentage of DCs and prognosis, the percentage of S100+ DCs was found to be lower in thymic carcinoma than in thymoma [5]. On the other hand, using fascin as a mature DC marker, DCs were reported to appear more frequently in benign thymic neoplasms [32]. In this study, we also confirmed the previous finding of a lower percentage of S100+ DCs in thymic carcinoma than in thymoma, indicating that malignant thymic epithelial tumor is associated with a paucity of DCs in their microenvironment.

Few studies examining the interaction between TAMs and DCs in malignant tumor are available. In Hodgkin’s lymphoma cases, a high percentage of TAMs and a low percentage of DCs were reported to be associated with adverse prognostic parameters [4]. In a comparison between benign and malignant skin tumors, malignant transformation of keratinocytes was found to be associated with infiltration of TAMs and loss of DCs [33]. Similarly, we found the same correlation, and this is the first report showing the association of thymic carcinoma with the increase in the percentage of TAMs and loss of DCs. Regarding the differentiation of monocytes, the CD115 (macrophage colony-stimulating factor receptor or CSF-1 receptor) pathway stimulates their survival and differentiation into macrophages rather than into DCs [34,35], and the CD115/CSF-1 pathway is reported to play a central role in tumor progression through its effects on the differentiation of TAMs [36]. Concerning the therapeutic aspects focusing on TAMs and DCs, recent studies revealed that some anti-CD115 monoclonal antibodies inhibit monocyte differentiation to TAMs, thereby skewing TAM differentiation toward DCs, and contributing to the generation of more efficient anti-tumor immune responses [37]. This study demonstrated an idea for future targeted therapeutic strategies for thymic carcinoma using such antibodies competing with CSF-1 binding to CD115.

## Conclusions

We examined the difference in the distribution profiles between TAMs and DCs in thymic epithelial tumors, and found a higher percentage of CD163+ TAMs and a lower percentage of S100+ DCs in thymic carcinoma tissues than in thymoma tissues. Our findings may provide an idea for future targeted therapeutic strategies for thymic carcinoma using antibodies that inhibit monocyte differentiation to TAMs, thereby skewing TAM differentiation toward DCs.
